# Cervical necrotizing fasciitis and acute mediastinitis of odontogenic origin: A case series

**DOI:** 10.4317/jced.53009

**Published:** 2017-01-01

**Authors:** Paolo Cariati, Fernando Monsalve-Iglesias, Almudena Cabello-Serrano, Alfredo Valencia-Laseca, Blas Garcia-Medina

**Affiliations:** 1 Oral and Maxillofacial surgery resident. Hospital Universitario Virgen de las nieves, Granada, Spain; 2 Maxillofacial Surgeon. Hospital Universitario Virgen de las nieves, Granada, Spain; 3 Oral and Maxillofacial Surgeon. Hospital Universitario Virgen de las nieves, Granada, Spain

## Abstract

Necrotising fasciitis (NF) is an uncommon infection. Early signs and symptoms include fever, severe pain and swelling, and redness at the wound site. Moreover, fulminant evolution and high mortality rate are typical of this pathology. In the present report we describes three cases of cervical necrotizing fasciitis complicated by acute mediastinitis. All patients were apparently immunocompetent adults. The main aim of the present report is to show the serious consequences that a dental infection might trigger. Furthermore, we highlight the importance of a multidisciplinary approach in these cases. The constant interaction between different medical specialties is essential for ensuring a proper management of each case.

** Key words:**Cervical necrotizing fasciitis, acute mediastinitis, odontogenic origin , multidisciplinary approach.

## Introduction

Necrotising fasciitis (NF) is an uncommoninfection of soft tissues caused by toxin-producing bacterias such as Group A beta-hemolytic Streptococcus pyogenes (GABHS) and Staphylococcus (1). It present two main features: fulminant evolution and high mortality rate (2). Perineum, extremities and abdominal region are typically the most affected by infection (3). In contrast, NF is less common in the head and neck area due to the high vascularization of cervical region (4). Despite this, the development of a cervical necrotizing fasciitis might be life-threatening (5). In fact, it is associated with mortality rates of 7% to 20% according to the extension of the infection at the time of diagnosis (3). In this line, is important to underline that diagnosis is frequently de-layed. The reason for this is that the onset of the disease may be accompanied by nonspecific symptoms of illness (6). Regarding the management, a multidisciplinary approach is mandatory. Early diagnosis, prompt surgical drainage, appropriate medical treatment and careful patient monitoring in an intensive care unit might improve the prognosis (7). The aim of the present report is to show the grave and serious consequences that a dental infection might provoke. Infection, cellulitis and abscess of dental origin should never be underestimated.

## Case Report

-Case 1: a 29 years old man consulted the emergency service of our hospital for tooth pain, swelling of the submandibular region and fever. The only important information that the patient referred was tooth pain (tooth 48) since four days. Patient exploration revealed macroscopic tooth decay of 48, trismus, floor of the mouth inflammation and diffuse pain on palpation of the submandibular zone. In view of that, intravenous antibiotic treatment was administered and a CT of cervical area was carried out. CT images showed the presence of a serious infection which spread from the tooth 48 to the posterior mediastinum. The patient was admitted in intensive care unit care unit of our hospital. A few hours later, thoracic and maxillofacial surgeons carried out the surgical drainage of the abscess. Moreover, we decided to performa surgical scrubbing of the infected area every 8 hours. In addition, a long treatment with intravenous broad-spectrum antibiotics was established. After surgery, the situation began to improve. Almost two months later, patient was discharged from the hospital.

-Case 2: a 43 years old man consulted the emergency service of our hospital for tooth pain and swelling of the right submandibular region. Patient referred that symptoms began two days before. Clinical exploration revealed trismus and hardening of the floor of the mouth tissues. A careful oral examination was hampered due to the intensive trismus. Thus, we decided to perform orthopantomography and a CT of cervical area in order to achieve a prompt diagnosis. Orthopantomography shows a wide tooth decay of 48. In this light, CT images showed a severe infection that spread from the tooth 48 to the posterior mediastinum. Against this backdrop, patient was admitted in intensive care unit care unit of our hospital. On the same night, a group of thoracic and maxillofacialsurgeons carried out the surgical drainage of the abscess. On this occasion we also performed the cleansing of the infected area every 8 hours. Moreover, a long treatment with intravenous broad-spectrum antibiotics was established. In the following days, patient evolved favorably and was discharged after almost forty-day of hospital stay.

**Figure 1:**
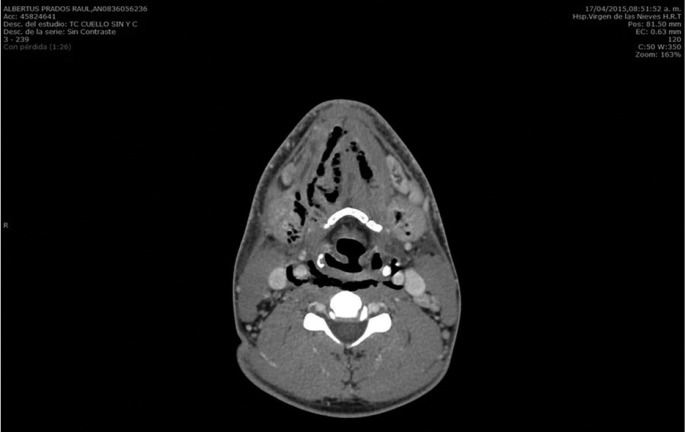
Air in the submandibular space, floor of the mouth and vascular space.

**Figure 2:**
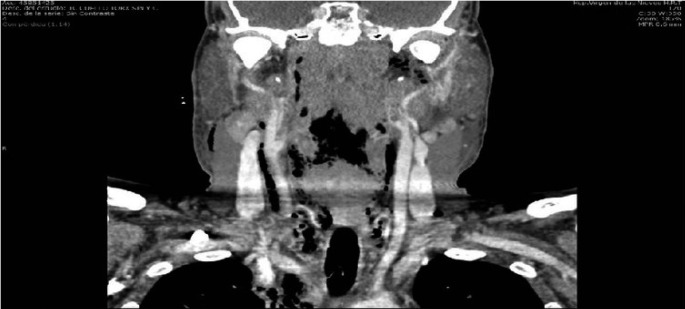
Dissection of cervical space due to toxins produced by bacteria.

**Figure 3:**
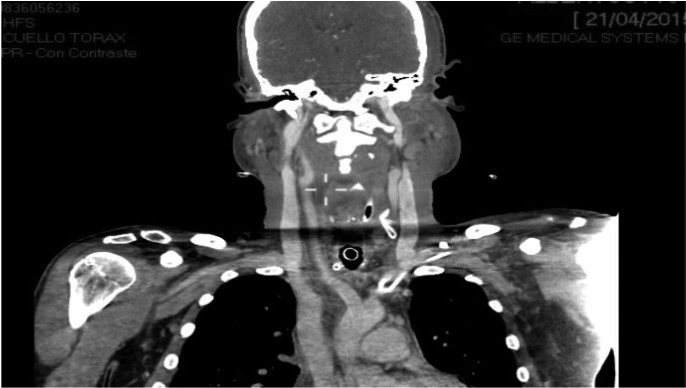
CT images that show the pus which spread from the tooth 48 to the mediastinum.

-Case 3: a 61 years old man consulted the emergency service of our hospital for tooth pain, trismus, swelling of bilateral parotid and submandibular region, dyspnea and hardening of the floor of the mouth tissues. Considering the above, an orthopantomography and a CT of cervical area were immediately carried out. This tests showed a grave infection that originated from the tooth 38. Importantly, CT images evidenced the presence of a large amount of air into parotid and submandibular region. Moreover, this test showed a collection of pus that extended from the tooth 48 to the posterior mediastinum. In the light of these developments, patient was admitted in intensive care unit care unit. Thoracic and maxillofacial surgeons carried out the surgical drainage of the abscess as quickly as possible. As before, we performed the cleansing of the infected area every 8 hours. Furthermore, a long treatment with intravenous broad-spectrum antibiotics was also established. Fortunately, infection evolved satisfactorily in this patient too. Approximately two months later, he was discharged from the hospital, (Figs. 1-3).

## Discussion

Cervical necrotizing fasciitis is a severe illness. In fact, when it is associated with descending necrotizing mediastinitis the rate of mortality amounts to 40% (8).

Diagnostic criteria for descending necrotizing mediastinitis are proposed by Estrera AS *et al.* (9). They are represented by.

A-Clinical manifestation of severe oropharyngeal infection.

B-Radiologic findings of mediastinitis on CT.

C- Intraoperative view of a necrotizing mediastinal infection or on postmortem examination.

D-Evident relationship between oropharyngeal infection and development.

Importantly, diabetes mellitus, chronic alcoholism, intravenous drug abuse, immunocompromised status, and obesity might represent a predisposing factor for the appearance of this pathology. In this line, we would like to highlight that our patient had none of the risk factors listed above. Indeed, they were apparently immunocompetent adults. The main aim of the present report is to show the serious consequences that a dental infection might provoke. Infection of dental origin require a proper assessment by specialized personnel. This infectious processes should never be underestimated. In addition, we firmly believe that a multidisciplinary approach in mandatory in these cases. The constant collaboration between different specialists is essential for ensuring a proper management of each cases. Critical care physicians, thoracic surgeons and maxillofacial surgeons must work together to make a correct diagnosis and treat the patient optimally. This report raises three central points. First, dental infection could trigger serious complication in certain circumstances. Second, an early diagnosis, prompt surgical drainage and appropriate medical treatment represent a key means of increasing patient survival. Third, adequate multidisciplinary support is imperative to ensure the proper management of these cases.
